# Epidemiology of Malaria, Schistosomiasis, Geohelminths, Anemia and Malnutrition in the Context of a Demographic Surveillance System in Northern Angola

**DOI:** 10.1371/journal.pone.0033189

**Published:** 2012-04-06

**Authors:** José Carlos Sousa-Figueiredo, Dina Gamboa, João Mário Pedro, Cláudia Fançony, António Justino Langa, Ricardo J. Soares Magalhães, J. Russell Stothard, Susana Vaz Nery

**Affiliations:** 1 Centre for Tropical and Infectious Diseases, Liverpool School of Tropical Medicine, Liverpool, United Kingdom; 2 Department of Infectious and Tropical Diseases, London School of Hygiene & Tropical Medicine, London, United Kingdom; 3 CISA Project (Health Research Center in Angola), Caxito, Bengo, Angola; 4 Escola Superior de Tecnologia da Saúde de Lisboa, Lisboa, Portugal; 5 School of Population Health, University of Queensland, Herston, Australia; Kenya Medical Research Institute - Wellcome Trust Research Programme, Kenya

## Abstract

**Background:**

Malaria, schistosomiasis and geohelminth infection are linked to maternal and child morbidity and mortality in sub-Saharan Africa. Knowing the prevalence levels of these infections is vital to guide governments towards the implementation of successful and cost-effective disease control initiatives.

**Methodology/Principal Findings:**

A cross-sectional study of 1,237 preschool children (0–5 year olds), 1,142 school-aged children (6–15 year olds) and 960 women (>15 year olds) was conducted to understand the distribution of malnutrition, anemia, malaria, schistosomiasis (intestinal and urinary) and geohelminths in a north-western province of Angola. We used a recent demographic surveillance system (DSS) database to select and recruit suitable households. Malnutrition was common among children (23.3% under-weight, 9.9% wasting and 32.2% stunting), and anemia was found to be a severe public health problem (i.e., >40%). Malaria prevalence was highest among preschool children reaching 20.2%. Micro-hematuria prevalence levels reached 10.0% of preschool children, 16.6% of school-aged children and 21.7% of mothers. Geohelminth infections were common, affecting 22.3% of preschool children, 31.6% of school-aged children and 28.0% of mothers.

**Conclusions:**

Here we report prevalence levels of malaria, schistosomiasis and geohelminths; all endemic in this poorly described area where a DSS has been recently established. Furthermore we found evidence that the studied infections are associated with the observed levels of anemia and malnutrition, which can justify the implementation of integrated interventions for the control of these diseases and morbidities.

## Introduction

Parasitic diseases in tropical regions are an important cause of morbidity and mortality. Malaria and neglected tropical diseases (NTD), such as schistosomiasis and geohelminths, incur enormous burdens on public health, mainly affecting impoverished rural communities characterized by poor sanitation and hygiene [1].

As malaria, schistosomiasis and geohelminths overlap geographically, i.e. cases of polyparasitism are common, there are synergistic interactions between diseases possibly exacerbating detrimental health consequences [2]. Whether or not helminth infections increase susceptibility to clinical/severe malaria is still debated and further research is needed [3]–[4]. The fact that there is overlap between malaria and NTDs, however, offers the opportunity for synergic strategies of disease control, specifically involving community health workers [5]–[6]. In fact, malaria control interventions would benefit greatly from the community-based approaches already in place for mass drug administration against NTDs [7].

Available ‘disease atlases’ for malaria and common NTDs are incomplete, with vast areas still to be studied (http://www.thiswormyworld.org/, http://www.map.ox.ac.uk/ and http://globalntddatabase.org/). One of these areas is Angola, in southern Africa, from which there is a definite lack of recently published information relating to the epidemiology and impact of malaria and NTDs.

Angola is estimated to have 3.4 million cases of malaria annually, mainly caused by *P. falciparum* [8]. Transmission occurs all year round, with greater seasonality in the south. Malaria is thought to be responsible for 35% of mortality in children under the age of five, 25% of maternal mortality, and 60% of hospital admissions for children under five [9]–[10]. A recent national malaria indicator survey found that 20% of children under five (reaching 29% in the hyperendemic area and a minimum of 6% in Luanda) and 14% of pregnant women (19% if in rural areas) were infected with *P. falciparum* [9]. National guidelines currently in place for malaria control include indoor residual spraying (IRS) in selected urban districts (covering over 100 000 households and 4% of the population at risk); free distribution of long-lasting insecticide-treated nets (LLITNs) at neonatal consultations, and free artimisinin-based combination therapy (ACT) at public health facilities (with over four million doses delivered in 2007–08, enough to treat almost 70% of the reported cases) [8].

Regarding geohelminths and schistosomiasis, a recent national survey was conducted to determine the National Neglected Tropical Disease guidelines in terms of mass drug administration (MDA) campaigns [11]. The observed cumulative prevalence of geohelminths and urinary schistosomiasis were 40% and 28%, respectively [11]. Despite the fact that these data call for mass-treatment of school-aged children against these NTDs (particularly in Northern and Central Angola) current interventions do not obtain the levels of efficacy necessary.

In 2007 the CISA project (*Centre for Health Research in Angola*, translated) was established as a result of a partnership between the Angolan Ministry of Health, the Bengo Provincial Government, the Portuguese Institute for Development Support and the Calouste Gulbenkian Foundation. CISA's Demographic Surveillance System (DSS) monitors over 60,000 people [12]., providing reliable information for the calculation of demographic indicators and facilitates the implementation of epidemiological studies.

The aim of this study was to determine the prevalence levels of (and associated variables for) malaria, schistosomiasis (urinary and intestinal), geohelminths, anemia and malnutrition in preschool (0–5 years old), school-aged (6–15 year old) children and their mothers/caregivers (16 and older) in rural and peri-urban areas in Dande Municipality (Bengo Province, Northern Angola), using CISA's DSS as the design and implementation platform. Information gathered during this study will augment previous work by government initiatives and provide data on concrete prevalence levels and associations between these infections, anemia and malnutrition.

## Methods

### The Demographic Surveillance System and study area

CISA's DSS was established in 2009, with the aim of collecting longitudinal data on the population's structure, dynamics and geographical location [12]. The DSS' study area includes three communes (Caxito, Mabubas and Úcua) within the Dande municipality, Bengo Province, north-western Angola, and is a largely rural area 60 km north of the capital Luanda. According to the census conducted between September 2009 and March 2010, this area of about 4,700 km^2^, has 60,075 registered inhabitants in 15,643 households distributed in 69 hamlets.

### Sample size calculation

Sample size calculation was based on estimates of prevalence levels (schistosomiasis) according to a recent national survey [11]. We estimated the population numbers necessary to conduct this survey with specified relative precision [13]. For schistosomiasis, taking into account a relative precision between 5–10% of the true prevalence (0.05<ε<0.10) and a reported prevalence of 21% (*P* = 0.21), we estimated that a sample size between 1537 and 6147 individuals would be sufficient. Due to logistics, we opted to aim for a sample size of 2835, well within both estimated intervals. Malaria and geohelminths were assumed to be more prevalent than schistosomiasis due to previously reported data (55% for malaria and 35% for geohelminths) [9], [11].

### Sample population and selection

This community-based cross-sectional epidemiological study took place between May and August 2010. The plan was to randomly select nine hamlets within each commune to a total of 27 hamlets.

From within each hamlet, 35 households would then be randomly selected from the DSS list of households that fulfilled the inclusion criteria: all households in each hamlet with at least one child aged 1 to 15 years and their mother/caregiver (16 to 49 years of age). The maximum goal was to reach 105 people per hamlet [ideally one preschool-aged child (0–5 years of age), one school-aged child (6–15 years of age) and their mother/caregiver per household], to a total of 945 individuals per commune and a maximum final number of 2835 individuals for the total survey area.

**Figure 1:**
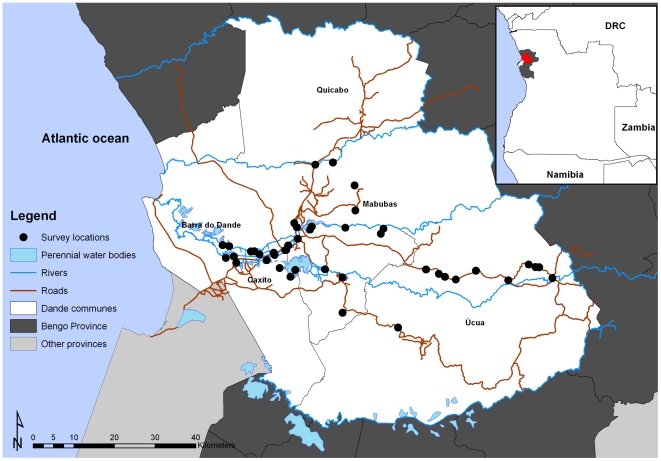
Map showing the location of the selected hamlets within the DSS area. The insert shows the location of the Bengo province (grey and red) and the communes of Caxito, Úcua and Mabubas (red), Angola.

To reach the necessary sample, the actual final number of hamlets included in the study was 9 in Caxito, 17 in Mabubas and 10 in Úcua. As a consequence, our actual sample size was larger than our target sample size: 36 v. 27 hamlets, 972 v. 945 households, 960 v. 945 mothers and 2379 v. 1890 children (1–15 year olds), respectively. The geographical location of the centroid of each of the 36 hamlets is shown in Figure 1.

### Questionnaire

Field workers were trained to interview caregivers using a questionnaire relating to the woman and her children, which recorded: demographic information (age, sex, literacy), occupation, access to healthcare and history of previous treatment, and questions related to malaria (e.g. bed-net ownership and utilization), schistosomiasis (e.g. water contact behavior and self-reported blood in urine) and geohelminth infections (e.g. having passed worms in stools and hand and food washing habits) (for a copy contact corresponding author).

### Measuring malnutrition and anemia

Each child was measured for height to the nearest 0.1 cm and for weight to the nearest 0.5 Kg. These measurements (along with age) were used to calculate an array of anthropometric indices used as proxies for malnutrition: weight-for-age (also known as under-weight); height-for-age (also known as stunting); weight-for-height (also known as wasting) and body mass index (BMI)-for-age (also known as thinness) [14]. Weight-for-age Z-scores were calculated for individuals 6 to 120 months of age (*N* = 1878); height-for-age and BMI-to-age Z-scores were calculated for individuals 6 to 240 months of age (*N* = 2426 and 2421, respectively), while weight-for-height Z-scores were calculated for individuals 6 to 60 months of age (*N* = 1046). For height-for-age and BMI-to-age Z-score calculation, individuals older than 15 years of age (i.e. female caregivers) were included in the analysis as the age restrictions allowed for this.

Results for all anthropometric indices were computed as Z-scores (number of standard deviations in relation to the mean of the standard population); the value of −2 Z-scores was used as the critical point below which to define malnutrition, while values bellow −3 Z-scores were defined as severe malnutrition. Z-scores were constructed using the 2006 World Health Organization (WHO; Geneva, Switzerland) database for child growth standards.

A finger prick blood sample was collected from each participant (caregivers and children). Hemoglobin (Hb) concentration was measured using a HemoCue® photometer (HemoCue 201+ system, HemoCue, Angelholm, Sweden). Anemia was diagnosed according to Hb thresholds set by WHO anemia [15]. Hb levels were not adjusted for altitude as all hamlets surveyed were below 1,000 meters [16].

### Parasitological techniques

Blood smears prepared in the field were later stained with 10% Giemsa and screened for malaria parasites by two independent microscope technicians (double-blind) [17].

A single stool and a single urine sample were collected from each child and caregiver participating in the study. The stool samples were used to prepare two Kato-Katz smears for intestinal schistosomiasis (*S. mansoni*), geohelminth infections (hookworm species – *Necator americanus, Ancylostoma duodenale -*, *Ascaris lumbricoides*, *Trichuris trichuria*), *Hymenolepsis nana* and *Taenia spp.* investigation [18]. Note that although not ideal for diagnosis of *Enterobius vermicularis*, microscope technicians were also asked to identify and register eggs from this roundworm. Slides were read (by two independent readers) between 30 min and 2 hours after processing. The results were expressed as eggs per gram (epg) of feces and infection intensities categorized according to WHO guidelines [19].

Micro-hematuria in urine was used as proxy-diagnosis of urinary schistosomiasis (*S. haematobium*), measured with urine-reagent strips (Hemastix®, Bayer, Leverkusen, Germany), an accepted marker in the rapid diagnosis of urinary schistosomiasis [20]. A positive diagnosis was considered for positive (single+and above) results only, i.e. not including traces.

### Statistical analysis

The questionnaire and lab results for each participant were double entered into a PostgreSQL® database system, transferred into Excel® for initial data check/cleaning and then imported to R statistical package® v 2.10.1 for statistical analysis. Note that the sample size for each variable will differ from the complete sample size, as 8 questionnaires were not properly completed and 282 stool samples (201 children and 81 mothers) and 193 urine samples (188 children and 5 mothers) were not handed-in by individuals.

For prevalence values, 95% confidence intervals (CI_95_) were estimated using the exact method [21]. Prevalence comparisons were performed using (one-tailed) Fisher's exact modification of the 2×2 chi-squared test.

For infection intensity values of parasites, the geometric mean of Williams, GM_W_, was chosen as the measure of central tendency due to the typical over-dispersion present in this type of data, and CI_95_ values for GM_W_ were estimated according to Kirkwood and Stern and used to estimate statistical difference between GM_W_ values [22]. When analyzing central tendency in normally distributed data (other than infections) a (trimmed) arithmetic mean was calculated.

In order to identify variables associated with infections and morbidities, multivariable models were developed. In these models, since children/mothers were from different hamlets, analysis took into account hamlet intra-correlation in the data using a generalized linear mixed model with multivariate normal random-effects (the random-effects of hamlet in our case), with penalized quasi-likelihood (function glmmPQL in R) [23]. Models were established defining affected children/mothers as cases, i.e. morbidity was treated as a binary variable (present and not present), and incorporating all variables. Forwards and backwards stepwise selection was performed to select the most parsimonious yet adequate final model using the Akaike information criterion (AIC) [24].

All models presented anemia controlled for sex (children) and age (children and mothers). The model for anemia in children included only children above two years of age, to avoid confounding by maternal Hb levels. For each variable, odds ratios (OR) and *P*-values were calculated, and a *P*-value <0.05 was considered indicative of statistical significance. These models were established in an attempt to identify statistical associations between the variables investigated during the survey.

### Ethical Approval, Treatment and Informed Consent

The study protocol was approved by the Angolan Ministry of Health Ethics Committee.

Participants found to have malaria (according to a rapid diagnostic test – Paracheck-Pf, Orchid, India) were treated with ACTs; those with *S. haematobium* (Hemastix positive) or *S.mansoni* infection (CCA positive) were treated with a standard dose of praziquantel (40 mg/Kg) and those found to have any geohelminth received 400 mg albendazole (children under two years of age were treated with 200 mg of albendazole).

One day before the survey, a field worker visited each household to provide explanations on the study and obtain informed signed consent (or fingerprint) from the selected caregiver. Each caregiver who had agreed to participate was given sample containers and informed how to collect fecal samples. All households that agreed to participate were enrolled in the study after handing in the signed consent form and the fecal samples.

## Results

### The population

A total of 972 households were selected and successfully included in the study, representing a total of 960 mothers (mean age 33.3 years, range 16 to 80 years) and 2,379 children (mean age 5.9 years, range 6 months to 15 years)). Forty one of the households included in the study did not have a suitable maternal figure, while 905 households had one complete family (mother and respective children), 23 had two families and 3 had three families. With a female to male ratio of 1.05 among the children, this survey included 1,157 boys and 1,222 girls aged 15 years or less. Of the 1,222 girls and 952 mothers/caregivers successfully interviewed, 3 (0.2%) and 134 (14.1%) reported being pregnant at the time of the survey, respectively.

### Anthropometric indices

A total of 23.3% of the individuals aged between 6 months and 10 years surveyed were found to be underweight (weight-for-age Z-score<−2). Stunting (height-for-age Z-score<−2) was highly prevalent in the population aged between 6 months and 20 years, reaching a prevalence of 32.2%. Wasting (weight-for-height Z-score<−2) prevalence reached 9.9% in children aged between 6 months and 5 years, and prevalence of thinness (BMI-for-age Z-score<−2) reached 10.7% of children aged between 6 months to 20 years. For CI_95_, prevalence levels according to sex and prevalence levels for severe malnutrition values (Z-score<−3), see Table 1.

**Table 1:** Prevalence of malnutrition within our population (stratified by sex) relating to each anthropometric index.

			Male			Female			Total	
Anthropometric measure (age range)	Value	Total (N)	No. (%)	CI_95_ (%)	Total (*N*)	No. (%)	CI_95_ (%)	Total (*N*)	No. (%)	CI_95_ (%)
Weight-for-age (6 to 120 months)	<−3 Z-score	914	85 (9.3)	7.5–11.4	964	58 (6.0)	4.6–7.7	1878	143 (7.6)	6.5–8.9
	<−2 Z-score		244 (26.7)	23.9–29.7		193 (20.0)	17.5–22.7		437 (23.3)	21.4–25.2
Height-for-age (6 to 240 months)	<−3 Z-score	1148	163 (14.2)	12.2–16.4	1278	104 (8.1)	6.7–9.8	2426	267 (11.0)	9.8–12.3
	<−2 Z-score		434 (37.8)	35.0–40.7		347 (27.2)	24.7–29.7		781 (32.2)	30.3–34.1
Weight-for-height (6 to 60 months)	<−3 Z-score	501	16 (3.2)	1.8–5.1	545	16 (2.9)	1.7–4.7	1046	32 (3.1)	2.1–4.3
	<−2 Z-score		58 (11.6)	8.9–14.7		46 (8.4)	6.2–11.1		104 (9.9)	8.2–11.9
BMI-for-age (6 to 240 months)	<−3 Z-score	1145	35 (3.1)	2.1–4.2	1276	35 (2.7)	1.9–3.8	2421	70 (2.9)	2.3–3.6
	<−2 Z-score		135 (11.8)	10.0–13.8		123 (9.6)	8.1–11.4		258 (10.7)	9.5–12.0
MUAC-for-age (6 to 60 months)	<−3 Z-score	441	3 (0.7)	0.1–2.0	457	1 (0.2)	0.0–1.2	898	4 (0.4)	0.1–1.1
	<−2 Z-score		11 (2.5)	1.3–4.4		12 (2.6)	1.4–4.5		23 (2.6)	1.6–3.8

SD stands for standard deviation, BMI stands for body mass index. Note that children with Z-scores of <−3 are a subset of children with value <−2, not a separate group; −2 Z-scores or lower identifies malnutrition, and −3 Z-scores or lower identified severe malnutrition.

After stepwise regression (*N* = 1625), girls (as compared to boys) were less likely to be underweight (OR = 0.62, CI_95_0.49–0.79, *P*<0.001), and children diagnosed with *A. lumbricoides* infection (OR = 1.36, CI_95_ 0.98–1.03, *P* = 0.063) and anemia (OR = 1.37, CI_95_ 1.07–1.76, *P* = 0.012) at the time of the survey were more likely to be underweight. Similarly, age (OR for every additional year = 0.92 CI_95_ 0.89–0.94, *P*<0.00001), sex (OR for girls compared to boys = 0.65, CI_95_ 0.54–0.78, *P*<0.00001) and anemia status (OR if anemic compared to negative = 1.59, CI_95_ 1.32–1.92, *P*<0.00001) at the time of the survey were included in the model for stunting (*N* = 2323). According to thinness, and after stepwise regression (*N* = 2180), older children (OR for every additional year = 1.04 CI_95_ 1.01–1.08, *P* = 0.008) and children diagnosed with *A. lumbricoides* infection at the time of the survey (OR = 1.53, CI_95_ 1.07–2.19, *P* = 0.019) were more likely to be acutely malnourished, while girls were less likely to be acutely malnourished than boys (OR = 0.70, CI_95_ 0.53–0.91, *P* = 0.009). Finally, neither age, sex, parasitic infection nor anemia were found to be statistically associated with wasting.

### Anemia

Anemia was found to be a severe public health problem, especially among children up to five years of age, where prevalence reached 56.9% and was found to be significantly higher than that in any other age-class (*P*<0.01) (Table 2).

**Table 2:** Anemia (stratified by age and sex) in three communes of the Bengo Province, Northern Angola.

	N	Mean Hb (SD) in g/L	Prevalence in % (and CI_95_) of anemia*
**Children (0.5–5 years)**	**1203**	**105.4 (15.5)**	**56.9 (54.0–59.7)**
Girls	619	106.8 (14.5)	51.5 (47.5–55.5)
Boys	584	104.0 (16.1)	62.5 (58.4–66.4)
**Children (6–12 years)**	**946**	**116.2 (14.1)**	**41.5 (38.4–44.8)**
Girls	464	115.9 (14.3)	41.6 (37.1–46.2)
Boys	482	116.4 (14.0)	41.5 (37.1–46.0)
**Teenagers (13–15 years)**	**169**	**121.5 (13.3)**	**43.8 (36.2–51.6)**
Girls	108	121.6 (11.4)	43.5 (34.0–53.4)
Boys	61	121.2 (16.2)	44.3 (31.5–57.6)
Women (pregnant)	131	110.1 (14.5)	44.3 (35.6–53.2)
Women (non-pregnant)	805	120.4 (14.5)	44.5 (41.0–48.0)

SD = standard deviation; CI_95_ = 95% confidence intervals;

* anemia was classified according to age, as described in Methods.

In children (2–15 years old), anemia was found to be associated with sex (girls less likely to be anemic than boys *P* = 0.032), increasing age (*P*<0.00001), *Plasmodium* spp. infection (*P*<0.00001), and micro-hematuria (*P* = 0.078) (see Table 3 for ORs and CI_95_).

**Table 3:** Model-fitting for anemia, malaria and urinary schistosomiasis, and geohelminths controlling for random-effects at the hamlet level.

Condition	Demographic group	Response variable	Baseline	Factor	Odds ratio (and CI_95_)	*P*-value
Anemia*	Children	Sex	Boy	Girl	0.82 (0.68–0.98)	0.032
		Age (continuous)		+1 year	0.92 (0.90–0.95)	<0.00001
		Malaria (microscopy	Negative	Positive	1.79 (1.37–2.32)	<0.00001
		Urinary schistosomiasis (Hemastix®)	Negative	Positive	1.30 (0.97–1.75)	0.078
Malaria	Children	Sex	Boy	Girl	0.82 (0.66–1.03)	0.082
		Age (continuous)		+1 year	1.01 (0.98–1.04)	0.61
		Mother knows malaria?	No	Yes	0.63 (0.47–0.85)	0.002
	Mothers	Age		+1 year	0.96 (0.94–0.98)	<0.001
		Pregnant	No	Yes	0.46 (0.24–0.90)	0.023
		History of previous treatment	No	Yes	0.60 (0.40–0.92)	0.020
*S. haematobium*	Children	Sex	Boy	Girl	0.99 (0.74–1.34)	0.97
		Age		+1 year	1.12 (1.07–1.16)	<0.00001
		Child bathes in river?	No	Yes	1.75 (1.06–2.91)	0.030
		Child bathes in dam?	No	Yes	20.23 (1.30–314.9)	0.032
	Mothers	Age		+1 year	0.99 (0.97–1.01)	0.20
		Pregnant?	No	Yes	0.56 (0.32–1.00)	0.049
		Self-reported water contact	Less frequently	Daily	2.08 (1.22–3.53)	0.007
STH infections*	Children	Age		+1 year	1.08 (1.06–1.11)	<0.00001
		Sex	Boy	Girl	0.90 (0.74–1.10)	0.313
		Self-reported worms in stool?	No	Yes	1.45 (1.17–1.80)	<0.001
		Self-reported tummy pain?	No	Yes	1.29 (0.96–1.74)	0.095

Anemia model included 1913 children >2 years of age, malaria model included 2309 children and 878 mothers, urinary schistosomiasis model included 2094 children and 894 mothers, and model for geohelminth infections included 2085 children.

* Note that none of the variables tested for mothers was significantly associated with anemia or with geohelminth infection.

### Malaria

Of all caregivers interviewed, 82.1% knew what malaria was, and close to half of all children (48.8% of preschool and 45.5% of school-aged children) and mothers (51.9%) interviewed had been treated for malaria in the past (therapeutic regimen unknown). Of the 134 mothers who reported being pregnant at the time of the survey, 44 (32.8%) also reported taking chemoprophylaxis against malaria during pregnancy. Bed-net coverage reached 25.1% of the interviewed families; and of those, 56.3% of preschool children, 44.5% of school-aged children and 60.7% of mothers reported having slept under the bed net during the last rains. On the other hand, a larger proportion of mothers reported having received larvicide for mosquito control (30.1%), most of whom reported using it frequently for treatment of water reservoirs in the household (93.7%).

*Plasmodium* spp. infection was significantly more prevalent among children (18.4% in preschool children and 18.2% in school-aged children) than in their mothers (9.6%, *P*<0.001) (Table 4). The geometric mean parasitaemia (of positives) in preschool children was significantly higher than that observed in school-aged children (649.9 parasites/µL v. 309.0 parasites/µL, *P*<0.001). A similar trend was found when comparing school-aged children and adults (309.0 parasites/µL v. 194.4 parasites/µL, respectively, *P* = 0.018). See Table 3 for other statistical associations.

**Table 4:** Prevalence (and CI_95_) of infections malaria, schistosomiasis and geohelminths.

	Preschool–aged children	School–aged children	Mothers
**No. of individuals recruited** *	1237	1142	960
**Malaria (Giemsa-stained microscopy)**	**18.4 (16.2–20.6)**	**18.2 (16.0–20.6)**	**9.6 (7.8–11.6)**
Light parasitaemia 1–499 parasites/µL of blood	50.2 (43.5–56.9)	70.2 (63.5–76.3)	81.5 (72.0–88.9)
Moderate parasitaemia 500–1,999 n/µL of blood	20.7 (15.6–26.6)	19.7 (14.5–25.8)	10.9 (5.3–19.0)
Heavy parasitaemia 2,000–9,999 n/µL of blood	19.8 (14.8–25.6)	6.7 (3.7–11.0)	4.3 (1.1–10.8)
Very heavy parasitaemia >10,000 n/µL of blood	9.3 (5.8–13.8)	3.4 (1.4–6.8)	3.3 (0.7–9.2)
Geometric mean of positives (CI_95_) in n/µL of blood	649.9 (648.6–651.1)	309 (307.7–310.2)	194.4 (193.1–195.7)
**Micro-hematuria (proxy for urinary schistosomiasis)**	**10.0 (8.2–11.9)**	**16.6 (14.5–18.9)**	**21.7 (19.1–24.4)**
***Ascaris lumbricoides*** **:**	**15.3 (13.2–17.6)**	**17.3 (15.1–19.7)**	**10.7 (8.7–12.9)**
Light parasite load 1–4,999 epg	9.4 (7.7–11.2)	10.5 (8.7–12.5)	7.7 (6.1–9.7)
Moderate to heavy parasite load ≥5,000 epg	5.9 (4.6–7.5)	6.8 (5.4–8.5)	3.0 (1.9–4.3)
Geometric mean (CI_95_) in epg	2.20 (1.02–3.38)	2.75 (1.55–3.95)	1.09 (0.00–2.25)
***Trichuris trichiura*** **:**	**7.2 (5.8–8.9)**	**13.9 (11.8–16.1)**	**9.7 (7.8–11.8)**
Light parasite load 1–999 epg	7.1 (5.7–8.8)	13.4 (11.4–15.6)	9.6 (7.7–11.7)
Moderate to heavy parasite load ≥1,000 epg	0.1 (0.0–0.5)	0.5 (0.2–1.1)	0.1 (0.0–0.6)
Geometric mean (CI_95_) in epg	0.37 (0.00–1.45)	0.90 (0.00–2.01)	0.52 (0.00–1.61)
**Hookworms (Kato–Katz)**	**4.2 (3.1–5.6)**	**6.7 (5.3–8.4)**	**13.7 (11.5–16.1)**
Light parasite load 1–1,999 epg	3.9 (2.8–5.2)	6.5 (5.1–8.1)	13.1 (10.9–15.5)
Moderate to heavy parasite load ≥2,000 epg	0.4 (0.1–0.9)	0.3 (0.1–0.8)	0.6 (0.2–1.3)
Geometric mean (CI_95_) in epg	0.24 (0.00–1.31)	0.42 (0.00–1.51)	1.02 (0.00–2.15)
**Other intestinal parasites (Kato–Katz)**			
*Enterobius vermicularis*	0.3 (0.1–0.8)	0.2 (0.0–0.7)	0.1 (0.0–0.6)
*Hymenolepsis nana*	6.2 (4.9–7.8)	7.3 (5.8–9.0)	1.9 (1.1–3.1)
*Taenia* spp.	0.1 (0.0–0.5)	0.2 (0.0–0.7)	0.1 (0.0–0.6)

Children were divided into two groups: preschool children (0–5 years of age) and school-aged children (6–15 years of age).

* Note that for the each diagnostic tool, the sample population size (*N*) was different; for information on number of tests conducted see Results.

### Schistosomiasis

Although knowledge of schistosomiasis (more specifically “disease that causes blood in urine” as in questionnaire) was relatively high among the studied population (64.8% of adults), reported history of previous treatment to this disease was very low (3.5% in preschool children, 5.9% in school-aged children and 5.5% in mothers). Notably, children who reported to be attending school at the time of the survey were more likely to have been treated for urinary schistosomiasis in the past (OR = 1.66, *P* = 0.02), but not mothers (*P* = 0.69).

The prevalence of micro-heamaturia was 10.0% in preschool children, 16.6% in school-aged children and 21.7% in mothers (significant difference in prevalence of infection between age-categories, *P*<0.01) (Table 4). For risk factors of urinary schistosomiasis, see Table 3. According to single sample microscopy, only two individuals were egg-patent for intestinal schistosomiasis (a five year-old girl with 36 epg and a 23 year-old mother with 132 epg).

### Geohelminths

Knowledge of geohelminth infections (referred to as *lombrigas*, the Portuguese layman's term for intestinal worms) was high among the studied population (85.4% of adults), and prevalence of previous treatment to this diseases was relatively low (15.9% in preschool children, 22.0% in school-aged children and 20.5% in mothers). Those who reported to be attending school at the time of the survey were more likely to have been treated for geohelminth infections in the past (OR for children = 1.44, *P*<0.001, OR for mothers = 1.49, *P* = 0.030).

The prevalence levels of being infected with at least one geohelminth infection were as follows: 22.6% (CI_95_ 20.2–25.2%) of preschool children, 31.6% (CI_95_ 28.9–34.5%) in school-aged children and 28.0% (CI_95_ 25.0–31.1%) (significant difference between child age-categories, *P*<0.01). Note that 3.8% (CI_95_ 2.7–5.1%) of preschool-aged children, 5.9% (CI_95_ 4.6–7.5) of school-aged children and 5.6% (CI_95_ 4.2–7.3%) of mothers were diagnosed with two or more geohelminth infections. For general prevalence levels of each infection, including information on infection intensities, see Table 4; for statistical associations see Table 3. For more information on statistical associations between individual geohelminths and the variables included in the questionnaire, please see Table S1.

## Discussion

This survey is one of the few comprehensive studies to be conducted in Angola (and made public) in the past forty years, relating to the epidemiology of malaria and NTDs, particularly involving such a large population. The fact that this population is being monitored by a DSS initiated by the CISA Project was crucial during planning and implementation of this study. Since both the local administration and the community were familiar with the DSS, community mobilization was facilitated. More importantly using the DSS database allowed for identification and selection of eligible households and participants for targeted visits to the communities, therefore increasing the efficiency of the survey and ensuring that participants were in fact residents of the area. Additionally, the DSS will be instrumental for future follow-ups in the context of a subsequent intervention study, evaluation of a governmental control initiative, or the establishment of subsequent cohort studies. Finally, as the DSS also collects information on socio-economic characteristics of the households, literacy, water sources and latrine utilization, and is coupled to a geographic information system, detailed risk and prediction maps can be developed.

### Malnutrition and anemia are public health problems

Malnutrition was highly prevalent in the studied population, supporting the fact that childhood malnutrition is persistent even under peaceful conditions, and must be targeted by governmental public health initiatives [25]. The prevalence values reported here for the anthropometric indices investigated are comparable to those reported by UNICEF for Sub-Saharan African, and more specifically for Angola [26]. Furthermore, two recent surveys conducted in Angola and involving a larger geographical scale report prevalence ranges comparable to those reported here for underweight (19.5% to 30.5%), stunting (19.5% to 45.2%) and wasting (6.3% to 6.9%) [11], [27].

Previous studies have found malnutrition in Angola to be associated with socio-economic status, where the poorest are more likely to be malnourished, independently of living in rural or urban settings, as well as geographical area, whereby children from areas where fighting during the civil war had been particularly ferocious and generalized, exhibited significantly higher levels of malnutrition, especially of stunting [27]–[29]. In this study, we have identified age, sex, anemia and soil-transmitted helminths, more particularly *A. lumbricoides*, to be significantly associated with the different indices of malnutrition. We cannot decipher the directionality of causality in the relationship between anemia and malnutrition, but these associations have been previously reported [30]–[32]. This is not the first time geohelminth infections, particularly *A. lumbricoides*, have been found to be significantly associated with acute malnutrition and being underweight, but identifying this association in Angola is new evidence to support policy change when targeting malnutrition [33]–[34].

Anemia was also found to be a public health problem among our study population with prevalence levels significantly higher than that reported by WHO for Angolan under-fives (56.9% v. 29.7%) [35]. The association between urinary schistosomiasis and anemia can be cryptic and hard to assess during cross-sectional surveys, nevertheless, it has been reported several times and should be considered when tackling anemia on a national scale [36]–[39]. Malaria, on the other hand, is considered to be the major infectious risk factor (perhaps evolutionary inducer) for the high prevalence of anemia in sub-Saharan Africa [40]–[41]. Targeted initiatives, such as free provision of anti-malarial treatment in point-of-care facilities, LLITN distribution and micronutrient supplementation can then lead to large improvements in prevalence of anemia (and thus malnutrition) [5], [42]. However, it is also important to consider the role of school- or community-based mass drug administration of praziquantel and albendazole targeting schistosomiasis and geohelminth infections, respectively, as these will also impact prevalence of anemia in a very cost-effective way [5]. These strategies could potentially be supplemented by implementation of WHO guidelines for short-term management of malnutrition, and ensure true long-term sustainable results [43].

### Malaria in this region of Angola

Prevalence of *Plasmodium* spp. infection among under-fives was 18.4%, 18.2% among school-aged children and 9.6% among the mothers. Pregnant women from our study population were less likely to have malaria than non-pregnant ones (OR = 0.46, *P* = 0.023); it is important to note that 32.8% of pregnant women were taking intermittent preventive treatment (bed-net coverage and bed-net use was equal between pregnant and non-pregnant women) [44]. The level of malaria among under-fives is much lower to that reported in 2007 by a government survey (28.8%) [9]. Surprisingly, our results show that under-fives are just as likely to have malaria as their older counterparts (6 to 15 year olds). Additionally, disease awareness and treatment seeking behavior were found to play important roles in reducing the infection risk for both mothers and children. These results demonstrate the importance of public health awareness campaigns; populations must be aware of the disease, must seek help when necessary and must know how to use bed-nets when they are provided. Otherwise, availability of suitable chemotherapy and bed-nets will not have the desired impact.

Of note is the fact that field-work was carried out between May and beginning of August, i.e. the end of the rainy season to beginning of dry season, which could lead to lower infection prevalence levels.

Even though first line prevention of malaria relies on nation-wide distribution of LLITN at antenatal consultations, we found surprisingly low bed-net coverage (25.1%), much lower in fact that what was reported in the 2007 Angola malaria indicator survey (53.8%), and especially worrying as Angola is benefiting from the President's Malaria Initiative since 2005 [9]–[10]. In fact, close to half of the households with bed-nets report having bought them and not having been provided by control initiatives.

### Schistosomiasis and Geohelminths

Although many mothers reported knowing of a disease that causes blood in urine (64.8%), very few reported ever receiving treatment for it (5.5%). The lack of preventive chemotherapy is reflected in the prevalence of micro-hematuria in both school-aged children (16.6%) and mothers (21.7%). These overall prevalence values were lower than the national average (28.0%), but still qualify for biannual MDA campaigns, according to WHO guidelines [11], [45].

The DSS area, specially Mabubas and Caxito communes, have a considerable number of water bodies – rivers, irrigation canals, ponds and a dam. Our analysis shows that children who regularly bathe in the river (OR = 4.72) and dam (OR = 22.23) were significantly more likely to be infected than those who did not.. The identification of the dam on the river Dande and the local ponds as transmission areas for urinary schistosomiasis warrants further mapping and should be the target of extensive malacological studies not only to pinpoint potential transmission hotspots but also to identify the intermediate host species of fresh water snail responsible for transmission in this area.

Geohelminth infections were common, being more prevalent among school-aged children. Overall, the level of geohelminth infections detected in our population was lower than the national average (40% school aged children) [11].). It is important to note, however, that our observed prevalence levels are likely to be an underestimation of the true levels as only a single stool was examined per individual [46]–[47]. Therefore, we argue that a secondary survey is warranted to assess more precisely prevalence levels of geohelminth infections, and provide further evidence for implementation of MDA campaigns in this area; that have been attempted previously by the national control program for NTDs albeit not regularly.

Infections with more than one geohelminth were not frequent (less than 6%), but interestingly, infection with one geohelminth increased the likelihood of infection with a second species. Multiple species parasite infections were present in around 5% of children and mothers for double infections with geohelminths and malaria or with geohelminths and schistosomiasis; whereas less than 2% of children and their mothers were simultaneously infected with malaria and schistosomiasis.

## Supporting Information

Table S1 **Model-fitting for each STH infection, controlling for random-effects at the hamlet level.**
(DOCX)
