# Supplementary material for: Epidemiology of Malaria, Schistosomiasis, Geohelminths, Anemia and Malnutrition in the Context of a Demographic Surveillance System in Northern Angola
Source: PLoS One. 2012 Apr 6;7(4):e33189. doi: 10.1371/journal.pone.0033189 (PMC3320883; doi:10.1371/journal.pone.0033189)
Supplement: Table S1 — Model-fitting for each STH infection, controlling for random-effects at the hamlet level. (DOCX) [file pone.0033189.s001.docx]

**Table S1 - Model-fitting for each STH infection, controlling for random-effects at the hamlet level**

| **Condition** | **Demographic group** |  | **Response variable** | **Baseline** | **Factor** |  | **Odds ratio (and CI_95_)** | ***P*-value** |
| --- | --- | --- | --- | --- | --- | --- | --- | --- |
|  |  |  |  |  |  |  |  |  |
| *A. lumbricoides* | Children |  | Age |  | +1 year |  | 1.01 (0.98–1.05) | 0.448 |
|  |  |  | Sex | Boy | Girl |  | 0.97 (0.76–1.24) | 0.820 |
|  |  |  | Self-reported worms in stool? | No | Yes |  | 1.63 (1.27–2.09) | <0.001 |
|  |  |  | Hookworms infection status | Negative | Positive |  | 1.97 (1.22–3.16) | 0.005 |
|  |  |  | *T. trichiura* infection status | Negative | Positive |  | 2.45 (1.74–3.44) | <0.00001 |
|  |  |  |  |  |  |  |  |  |
|  | Mothers |  | Age |  | +1 year |  | 0.97 (0.94–0.99) | 0.002 |
|  |  |  | Self-reported eczema in past? | Negative | Positive |  | 3.06 (1.36–6.88) | 0.007 |
|  |  |  | Hookworms infection status | Negative | Positive |  | 1.85 (1.06–3.22) | 0.031 |
|  |  |  | *T. trichiura* infection status | Negative | Positive |  | 3.02 (1.73–5.27) | <0.001 |
|  |  |  |  |  |  |  |  |  |
| *T. trichiura* | Children |  | Age |  | +1 year |  | 1.11 (1.07–1.15) | <0.00001 |
|  |  |  | Sex | Boy | Girl |  | 0.92 (0.70–1.20) | 0.525 |
|  |  |  | Hookworms infection status | Negative | Positive |  | 1.84 (1.12–3.00) | 0.016 |
|  |  |  | *A. lumbricoides* infection status | Negative | Positive |  | 2.43 (1.75–3.37) | <0.00001 |
|  | Mothers |  | Age |  | +1 year |  | 0.99 (0.97–1.01) | 0.26 |
|  |  |  | *A. lumbricoides* infection status | Negative | Positive |  | 3.27 (1.89–5.66) | <0.00001 |
|  |  |  |  |  |  |  |  |  |
| *Hookworms* | Children |  | Age |  | +1 year |  | 1.12 (1.07–1.17) | <0.00001 |
|  |  |  | Sex | Boy | Girl |  | 0.73 (0.51–1.04) | 0.080 |
|  |  |  | Mother knows about STHs | No | Yes |  | 0.57 (0.35–0.92) | 0.023 |
|  |  |  | *T. trichiura* infection status | Negative | Positive |  | 1.93 (1.21–3.10) | 0.006 |
|  |  |  | *A. lumbricoides* infection status | Negative | Positive |  | 2.07 (1.33–3.23) | 0.001 |
|  | Mothers |  | Age |  | +1 year |  | 1.01 (0.99–1.03) | 0.34 |
|  |  |  | Goes to school? | No | Yes |  | 0.44 (0.23–0.83) | 0.011 |
|  |  |  | *A. lumbricoides* infection status | Negative | Positive |  | 1.89 (1.07–3.35) | 0.028 |
|  |  |  |  |  |  |  |  |  |

Model for *Ascaris lumbricoides* included 2093 children and 830 mothers, model for *Trichuris trichiura* included 2139 children and 844 mothers and model for hookworms included 2110 children and 843 mothers.
